# Supplementary material for: Influence of Copper Valence in CuOx/TiO2 Catalysts on the Selectivity of Carbon Dioxide Photocatalytic Reduction Products
Source: Nanomaterials (Basel). 2024 Nov 29;14(23):1930. doi: 10.3390/nano14231930 (PMC11643810; doi:10.3390/nano14231930)
Supplement: Supplementary file 1 [file nanomaterials-14-01930-s001.zip › nanomaterials-3275887-supplementary.pdf]

Influence of copper valence in CuO_x/TiO_2 catalysts on the selectivity of carbon dioxide photocatalytic reduction products

Sha Ni, Wenjing Wu, Zichao Yang, Min Zhang, Jianjun Yang*

National & Local Joint Engineering Research Center for Applied Technology of Hybrid Nanomaterials, Henan University, Kaifeng, China

***Corresponding author: e-mail yangjianjun@henu.edu.cn**

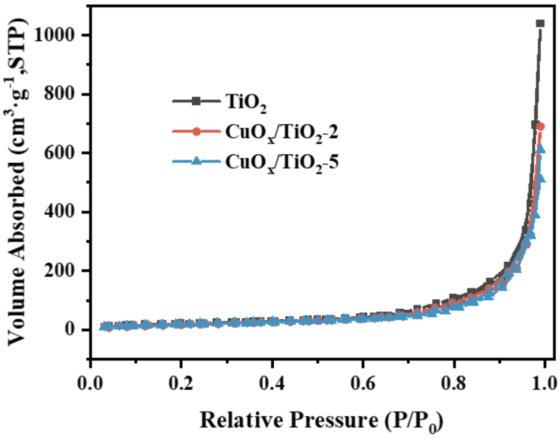

Figure S1. N_2 adsorption and desorption isotherms of TiO_2, CuO_x/TiO_2-2 and CuO_x/TiO_2-5.

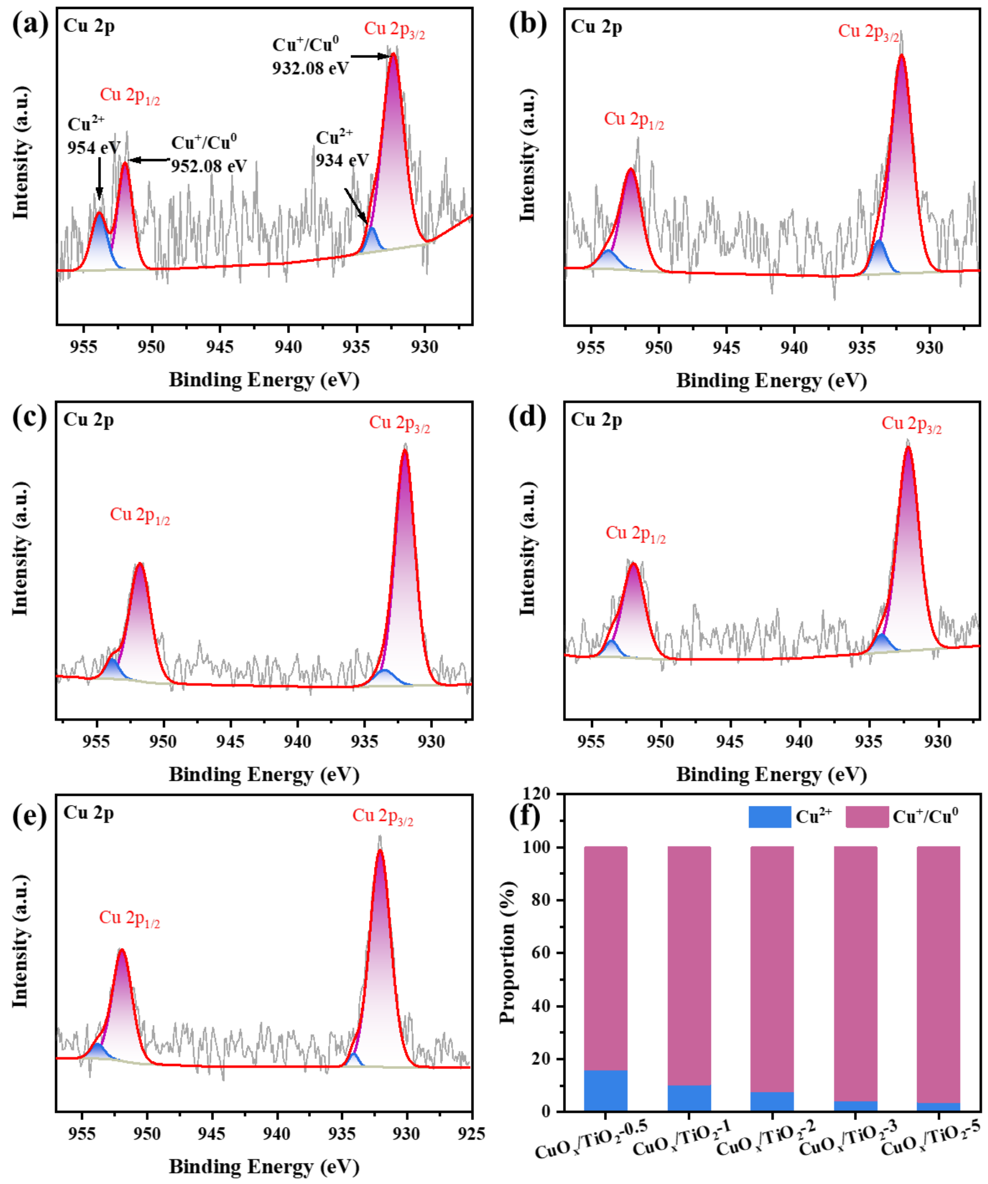

Figure S2. XPS of Cu2p spectra (a-e) CuO_x/TiO_2-y (y = 0.5, 1, 2, 3, 5), (f) Proportion of different state of Cu^{2+} and Cu^{+}/Cu^{0} components in CuO_x/TiO_2.

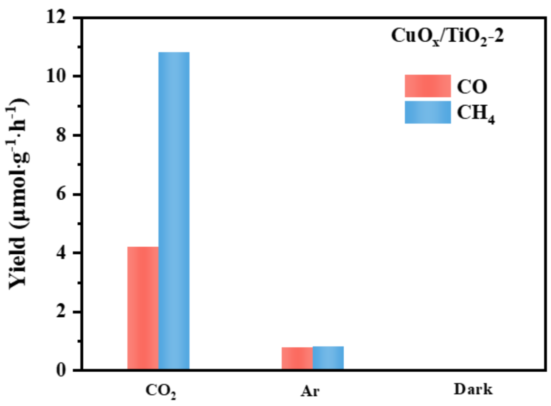

Figure S3. Photocatalytic CO_2 reduction activity of CuO_x/TiO_2-2 under different reaction conditions.
